# Oral Contraceptive Pills as a Potential Cause of Wallenberg Syndrome: A Mysterious Case

**DOI:** 10.7759/cureus.67733

**Published:** 2024-08-25

**Authors:** Anshul Ramanathan, Gretchen Junko

**Affiliations:** 1 Internal Medicine, Edward Via College of Osteopathic Medicine, Blacksburg, USA; 2 Internal Medicine, LewisGale Medical Center, Roanoke, USA

**Keywords:** ischemic stroke, embolic stroke of undetermined source, cryptogenic stroke, wallenberg syndrome, oral contraceptives

## Abstract

Wallenberg syndrome, also known as lateral medullary syndrome, is a rare neurological condition caused by an ischemic stroke in the posterior inferior cerebellar artery (PICA) territory of the brainstem. Here, we present a case of Wallenberg syndrome in a relatively healthy 37-year-old woman with no known risk factors besides a history of long-term oral contraceptive pill (OCP) use without prior adverse effects. The patient presented with acute onset headache that worsened in bright light, left-sided lightheadedness, dizziness, blurry vision, and non-bloody, non-bilious emesis. A neurological exam revealed left facial numbness and left upper extremity numbness; however, strength was intact in all extremities. An MRI revealed an acute ischemic infarct in the left PICA distribution, consistent with Wallenberg syndrome. While the initial thought was that the patient's OCP use contributed to this stroke, it has been deduced that the risk of stroke with current formulations of OCPs is insignificant compared to women who do not take any OCPs. This case highlights the importance of reconsidering OCPs as the cause of stroke in young, healthy patients without significant risk factors and considering reclassification as an embolic stroke of undetermined source (ESUS).

## Introduction

Posterior circulation strokes comprise 20% of all ischemic strokes [1]. Posterior circulation is defined by the territory supplied by the vertebrobasilar arterial system [1]. Wallenberg syndrome (lateral medullary syndrome) is a result of ischemic occlusion of the posterior inferior cerebellar artery (PICA). PICA originates from the vertebral artery and supplies the medulla [2]. The trunk of the PICA has five different branches that can supply different areas within the medulla [3].

Within the medulla lie the cerebellar peduncles, cranial nerves V, VIII, IX, and X, the spinothalamic tract, and sympathetic fibers. Occlusion of the PICA will lead to changes associated with knocking out the aforementioned structures and nerves. Symptoms of Wallenberg syndrome include vertigo, nausea, vomiting, ipsilateral facial numbness, Horner syndrome, dysphagia, and ataxia [1].

Common risk factors for stroke include hypertension, atrial fibrillation, hyperlipidemia, diabetes, smoking, and alcohol use. Some rare risk factors for stroke include hypercoagulable blood disorders (antiphospholipid syndrome, factor V Leiden, protein C/S deficiency), as well as genetic disorders such as cerebral autosomal dominant arteriopathy with subcortical infarcts (CADSIL), moyamoya disease, Ehlers-Danlos syndrome type IV, Fabry disease, and mitochondrial encephalomyopathy with lactic acidosis and stroke-like episodes (MELAS) syndrome. In addition to all these causes, it is well documented that oral contraceptive pills (OCPs) can lead to a hypercoagulable state given the nature of ethinyl estradiol. However, it is important to note that the stroke risk associated with OCP use is additive with other stroke risk factors, such as smoking and hypertension [4]. Here, we present a case of a healthy 37-year-old female without any risk factors besides a long-term history of OCP use who presented with an embolic stroke in the distribution of PICA, leading to Wallenberg syndrome.

## Case presentation

A 37-year-old female with a history of asthma and depression presented to the Emergency Department with acute onset of left-sided headache that worsened in bright light, lightheadedness, dizziness, blurry vision, and non-bloody, non-bilious emesis. On arrival at the ED, the patient had a BP of 223/112 mmHg, which improved with diazepam and meclizine. The patient continued to experience nausea and dizziness and reported new-onset left-sided facial numbness. The patient denied any significant medical history, specifically vertigo, migraines, or strokes. She denied any family history of brain aneurysms or cerebrovascular accidents. She denied any alcohol or tobacco use but endorsed occasional marijuana use. She had been taking low-dose oral contraceptives since she was 15 years old, without any complications.

A CT head and CT angiography of the head and neck were negative for any acute processes, including vertebral artery dissection. The patient was evaluated by tele-neurology, which confirmed a National Institutes of Health Stroke Score (NIHSS) of 1. Neurology also recommended admission for observation and MRI. The patient was subsequently admitted to the medicine floor.

**Figure 1:**
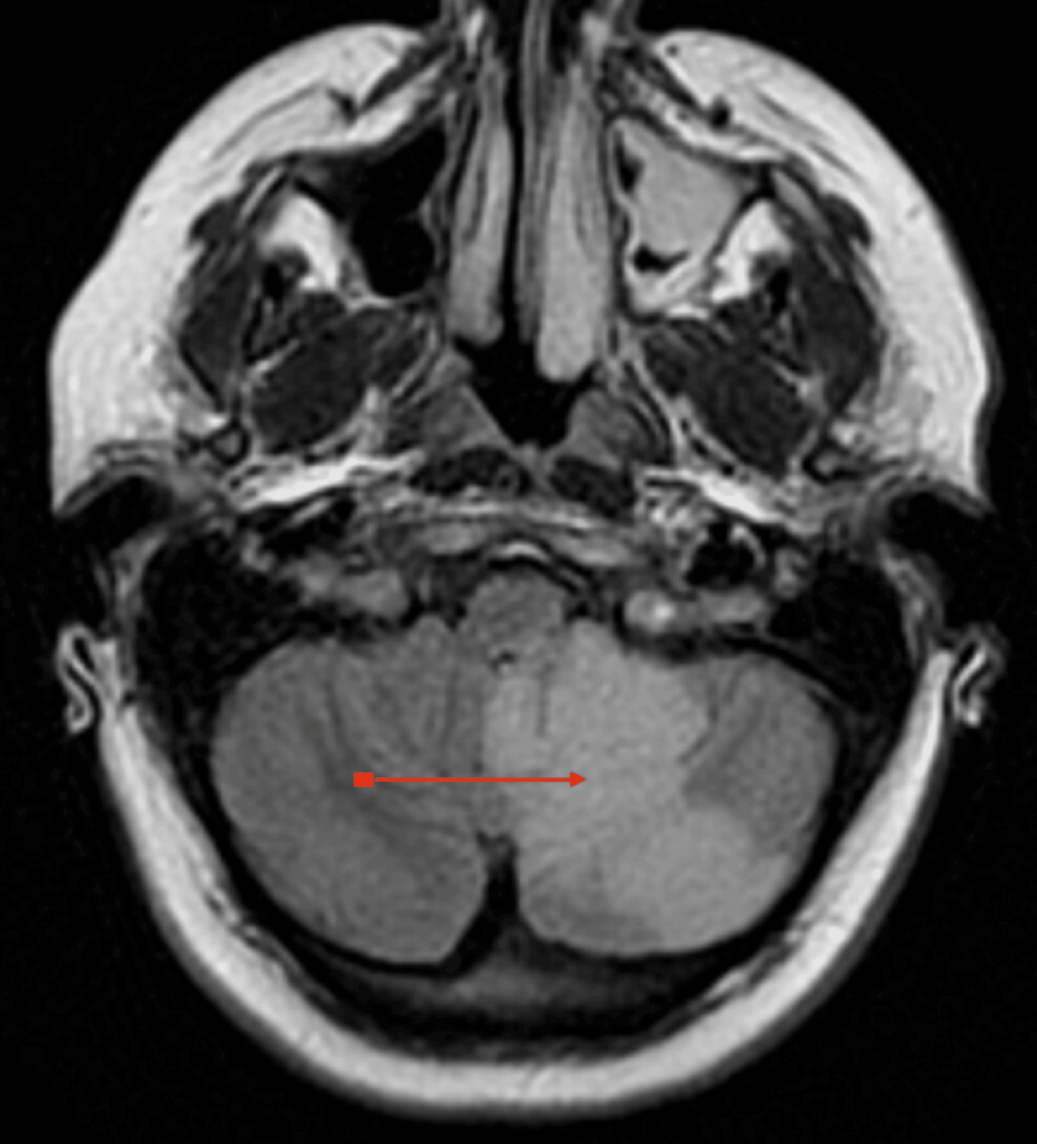
MRI of the patient's brain The red arrow depicts the area of the medulla where there is ischemia.

The patient was started on aspirin and atorvastatin. A lipid panel and A1C were ordered, both within normal limits. A migraine cocktail was ordered due to the patient's continued nausea, dizziness, and headache. Due to continued concerns for acute ischemic stroke, a permissive hypertension treatment strategy with 0.1 mg of clonidine every six hours was given with a goal blood pressure of 220/120 mmHg. A brain MRI was ordered which revealed a new acute ischemic infarct in the left PICA distribution concerning Wallenberg syndrome (Figure 1). MRI also revealed a slight midline shift with some brain swelling. Mild compression of the fourth ventricle without hydrocephalus was also noted. Neurosurgery was consulted and recommended ICU admission with every two hours of neuro checks. She was promptly started on dexamethasone (Decadron; Merck & Co., Inc., Rahway, NJ, USA) taper and hypertonic saline to reduce brain swelling.

The patient was noted to have hypertensive urgency and was placed on a nicardipine drip. Repeated head CTs were negative for hemorrhagic conversion. Eventually, hypertonic saline was discontinued, and the patient was downgraded to med-surg. A full work-up for the patient was ordered to elucidate the cause of the stroke. The chemistry profile was unremarkable. The hematology profile was mostly unremarkable, except for elevations in WBC (22.10), RBC (5.23), and mean platelet volume (MPV) (9.5 L). Immunological profiles measuring cytoplasmic anti-neutrophil cytoplasmic antibody (c-ANCA), proteinase 3 (PR3) antibody, atypical perinuclear anti-neutrophil cytoplasmic antibody (p-ANCA), anti-myeloperoxidase, and double-stranded DNA antibody were all within normal limits, which ruled out any vasculopathy. Coagulation profiles, including factor V levels, were all within normal limits, which ruled out any coagulopathies. Viral serology was unremarkable. The patient followed up with Cardiology in June. A transesophageal echo showed no evidence of thrombus in the left or right atrial cavity or appendage. No atrial septum defect was identified, and there were no signs of intracardiac mass, thrombus, or pericardial effusion. The patient was monitored with a Holter monitor for 2 days and 17 hours, which revealed a predominantly normal sinus rhythm.

## Discussion

Although it is well-documented that oral contraceptives can lead to a hypercoagulable state, current OCP formulations contain less than 50 micrograms of ethinyl estradiol [4]. Moreover, recent large-scale studies from a Swedish cohort have elucidated that, when adjusting for various risk factors such as smoking status, hypertension, diabetes, and alcohol use, there was no significant association between stroke and OCP use [4]. A meta-analysis evaluating combination OCPs, which controlled for smoking and hypertension, found a significantly lower risk of ischemic stroke - a relative risk of 1.93 (95% confidence interval: 1.35-2.74) [5]. In this patient’s case, her stroke was initially deduced to be due to her use of oral contraceptives, given the absence of other risk factors. Given that this healthy patient had been using low-dose OCPs since she was a teenager, had no history of hypertension or migraines, and did not smoke, it is unlikely that her current stroke is related to her OCP use. Her lack of significant risk factors characterizes her stroke as a cryptogenic stroke or an embolic stroke of undetermined source (ESUS).

ESUS represents approximately 17% of all ischemic strokes [6]. To meet diagnostic criteria for ESUS, ischemic strokes must meet four different criteria: strokes must be non-lacunar ischemic strokes as seen on CT or MRI; have an absence of atherosclerosis causing greater than 50% luminal stenosis in arteries supplying the ischemic area; no major cardioembolic source; and no other specific cause of stroke identified. This patient met all four criteria to classify her stroke as ESUS. Patients with ESUS tend to have less significant strokes, with average NIHSS ratings of 5 [6]. Pooled analysis revealed that the average age for ESUS is 65, and 42% were women [7]. This patient’s case is unique, given her younger age.

## Conclusions

Wallenberg syndrome is a stroke in the PICA. While OCP use has traditionally been considered a risk factor for strokes, recent studies have shown that the risk of stroke in those who take OCPs, compared to those who do not, is not statistically significant. In a healthy patient who uses low-dose OCPs but lacks atherosclerotic plaques, cardiogenic emboli, and other stroke risk factors, a stroke would be better classified as an ESUS. A deeper dive into the specific brand of OCPs that this patient was taking at the time of her stroke, or testing for hypercoagulable conditions such as malignancy, could help elucidate potential sources of the ischemia.
